# The Role of Ongoing Dendritic Oscillations in Single-Neuron Dynamics

**DOI:** 10.1371/journal.pcbi.1000493

**Published:** 2009-09-04

**Authors:** Michiel W. H. Remme, Máté Lengyel, Boris S. Gutkin

**Affiliations:** 1 Group for Neural Theory, Département d'Études Cognitives, École Normale Supérieure, Paris, France; 2 Computational and Biological Learning Lab, Department of Engineering, University of Cambridge, Cambridge, United Kingdom; 3 Group for Neural Theory, Département d'Études Cognitives, École Normale Supérieure, Paris, France; University College London, United Kingdom

## Abstract

The dendritic tree contributes significantly to the elementary computations a neuron performs while converting its synaptic inputs into action potential output. Traditionally, these computations have been characterized as both temporally and spatially localized. Under this localist account, neurons compute near-instantaneous mappings from their current input to their current output, brought about by somatic summation of dendritic contributions that are generated in functionally segregated compartments. However, recent evidence about the presence of oscillations in dendrites suggests a qualitatively different mode of operation: the instantaneous phase of such oscillations can depend on a long history of inputs, and under appropriate conditions, even dendritic oscillators that are remote may interact through synchronization. Here, we develop a mathematical framework to analyze the interactions of local dendritic oscillations and the way these interactions influence single cell computations. Combining weakly coupled oscillator methods with cable theoretic arguments, we derive phase-locking states for multiple oscillating dendritic compartments. We characterize how the phase-locking properties depend on key parameters of the oscillating dendrite: the electrotonic properties of the (active) dendritic segment, and the intrinsic properties of the dendritic oscillators. As a direct consequence, we show how input to the dendrites can modulate phase-locking behavior and hence global dendritic coherence. In turn, dendritic coherence is able to gate the integration and propagation of synaptic signals to the soma, ultimately leading to an effective control of somatic spike generation. Our results suggest that dendritic oscillations enable the dendritic tree to operate on more global temporal and spatial scales than previously thought; notably that local dendritic activity may be a mechanism for generating on-going whole-cell voltage oscillations.

## Author Summary

A central issue in biology is how local processes yield global consequences. This is especially relevant for neurons since these spatially extended cells process local synaptic inputs to generate global action potential output. The dendritic tree of a neuron, which receives most of the inputs, expresses ion channels that can generate nonlinear dynamics. A prominent phenomenon resulting from such ion channels are voltage oscillations. The distribution of the active membrane channels throughout the cell is often highly non-uniform. This can turn the dendritic tree into a network of sparsely spaced local oscillators. Here we analyze whether local dendritic oscillators can produce cell-wide voltage oscillations. Our mathematical theory shows that indeed even when the dendritic oscillators are weakly coupled, they lock their phases and give global oscillations. We show how the biophysical properties of the dendrites determine the global locking and how it can be controlled by synaptic inputs. As a consequence of global locking, even individual synaptic inputs can affect the timing of action potentials. In fact, dendrites locking in synchrony can lead to sustained firing of the cell. We show that dendritic trees can be bistable, with dendrites locking in either synchrony or asynchrony, which may provide a novel mechanism for single cell-based memory.

## Introduction

The dendritic tree contributes significantly to the elementary computations a neuron can perform, both by its intricate morphology and its composition of voltage-gated ionic conductances [1]. Such active conductances can underlie a wide variety of dynamical behaviors, amongst others dendritic spikes and ongoing oscillations of the dendritic membrane potential [2]. Such active dendritic phenomena have been suggested as mechanisms endowing single neurons with significant computational power [3] and flexibility in the way the dendritic tree processes its inputs: whether as a global element, effectively collapsing the tree into a single functional compartment or with various parts of the tree acting as independent processing elements [4],[5]. While the possibility of powerful and flexible dendritic processing has been of great interest, the precise conditions under which dendrites can act independently or globally remain largely to be determined. In this report we address this key question, focusing specifically on the case where active properties lead to sustained intrinsic membrane potential oscillations in the dendrites. We develop a theoretical formalism, allowing for a succinct yet powerful description of the dendritic tree dynamics and yielding conditions under which the tree acts as a global oscillatory unit and how such action in turn controls spiking responses of the neuron.

Membrane potential oscillations have been demonstrated in various types of neurons. Prominent intrinsic subthreshold oscillations have been found in stellate cells from entorhinal cortex layer 2 [6],[7], neurons from the frontal cortex [8], neurons from the amygdala complex [9],[10], and pyramidal cells and interneurons from the hippocampal CA1 area [11],[12]. Although these membrane potential oscillations are normally recorded at the soma and thus are considered to be of somatic origin, several lines of evidence suggest dendritic loci of generation. First, many of the conductances thought to underlie the generation of such oscillations reside predominantly in the dendrites, sometimes specifically in the distal parts of the dendritic tree. For example, in the apical dendrites of hippocampal CA1 pyramidal neurons, the density of 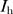 increases strongly with distance from the soma [13], and reaches very high values in the thin distal branches [14]. Second, several studies have suggested the existence of clusters of ionic conductances that are responsible for the generation of dendritic spikes [15]. While most of the direct electrophysiological evidence regards excitable behavior, demonstrating the generation of dendritic spikes in response to sufficient levels of depolarization, mathematical analysis has shown that neural membranes exhibiting excitability can readily pass to oscillatory regimes in an input-dependent manner (e.g. see [16]). Third, in several cases, oscillations have been directly recorded in dendrites. For example, recordings from hippocampal CA1 pyramidal neurons have demonstrated ongoing oscillations in the dendrites that include repetitive dendritic spikes, presumably involving Ca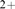 currents [17]. Furthermore, significant intrinsic dendritic oscillations have been observed in several neuronal preparations that depended on the interplay between the non-linear properties of NMDA synaptic receptors and intrinsic voltage-dependent currents [18],[19]. Crucially, while the onset of these oscillations was conditional on the activation of the NMDA synapses, the oscillations themselves were produced by mechanisms that were intrinsic to the postsynaptic cell and not by periodically structured synaptic inputs. Since NMDA receptors are largely localized on dendritic spines, and are hence electrotonically removed from the soma, these data may also argue for a non-uniform and local dendritic generation of membrane potential oscillations. Taken together, these experimental results suggest that dendritic trees can function as oscillators, perhaps conditional on the level of background depolarization or the presence of neuromodulators [20], while leaving open the question whether global cell-wide voltage oscillations could result from local dendritic mechanisms that are intrinsic even to distal dendrites and hence only weakly coupled to the soma electrotonically.

Indeed, multiple intrinsic dendritic oscillators have been proposed to underlie the recently discovered intricate firing pattern of entorhinal grid cells [21]–[23]. This influential model suggests that the functional responses of entorhinal neurons recorded in behaving animals are a direct consequence of the generation of independent oscillations that are intrinsic to individual dendrites. Hence, this model presupposes the existence of multiple oscillators that are integrated at the soma, leading to the questions of how such dendritic oscillators may interact with the soma and with each other, and what sorts of collective behaviors the electrotonic structure of the dendritic tree might impose on the oscillations.

In this paper, we study the dynamics of such interacting oscillators and their impact on signal propagation in single neurons, using mathematical analysis corroborated by numerical simulations of biophysical models. We treat the dendritic tree of a neuron as a network of oscillators coupled by stretches of relatively less active cable. This prompts us to combine two analytical methods: weakly coupled oscillator theory and cable theory. The theory of weakly coupled oscillators has been extensively used previously to study synchronization of multiple oscillators residing in separate cells interacting through synapses or gap junctions [24]. Since we focus on intradendritic oscillators which are continuously coupled via the membrane voltage, we use cable theory [25] to compute their interactions.

We find that intradendritic oscillations can exhibit complex patterns of phase-locking. We characterize how this phase-locking depends on the intrinsic properties of the oscillators and on the membrane properties of the segment connecting them. Finally, we demonstrate how input to the dendritic oscillators can control the phase-locking and how in turn the phase-locked configuration can control somatic spike generation. These results provide a rigorous mathematical framework for the study of interacting dendritic oscillations that can be applied in the future to specific systems of interest, and also point to ways in which such oscillations can be utilized for non-trivial single cell computations.

## Results

Our goal is to develop a theory for the behavior of a dendritic tree that contains multiple intrinsic oscillators and then use this framework to gain understanding of how such a tree would behave dynamically and hence control the neuron's output depending on the input. In order to develop the mathematical framework we begin by considering a minimal setup of two cable-coupled oscillators. As we will see even this setup is too complicated for direct analytical treatment hence we will go through a number of reduction steps which we sketch out below.

**Figure 1:**
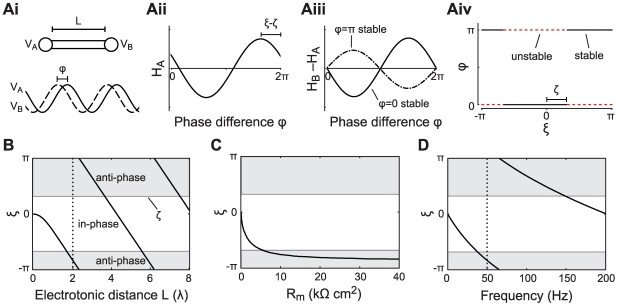
Passive cable coupling. Ai: The oscillators with voltage trajectories 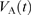 and 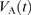 and phase difference 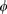 determine the membrane potential at the ends of a cable with electrotonic length 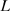. Aii: The interaction function 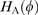 gives the phase shift of oscillator A as a function of 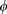. This interaction function is shifted along the 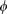-axis by the parameters 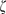 and 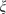, which capture the oscillator and cable properties, respectively. Aiii: The stable phase-locked solution is determined by 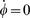 and 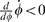 and is either at 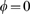 (e.g. for the solid curve) or at 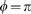 (e.g. for the dash-dotted curve). Aiv: The stable phase-locked solution as a function of 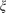. The value of 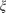 uniquely determines where the in-phase (black solid line) or the anti-phase solution (red dotted line) is stable, given a fixed value of 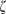. B: 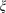 as a function of the electrotonic distance 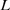 between the oscillators, 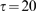 ms and 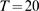 ms (dotted line in panel D). For illustrative purposes we chose 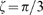 so that the stable in-phase and anti-phase solutions are given by the white and gray areas, respectively. C: 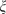 as a function of the membrane resistance 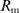 for cable diameter 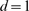 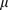
m, distance between the oscillators 1000 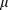
m, membrane capacitance 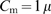
F/cm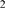, intracellular resistivity 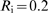 k
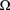
cm and oscillator period 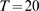 ms. D: 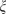 as a function of the oscillator frequency 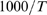. The distance between the oscillators is 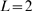 (dotted line in B), 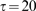 ms.

We study the behavior of a system of two oscillators with period 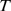 being connected via an active (though not intrinsically oscillating) dendritic cable with length constant 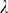 and membrane time constant 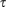. The two oscillators A and B are located at the ends of the cable at 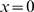 and 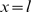, separated by an electrotonic distance 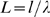 (figure 1Ai). In general form the system we will consider for describing the membrane potential 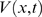 along the dendritic cable is given by the following equations: 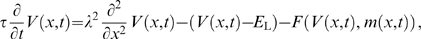
(1)

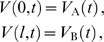
(2)

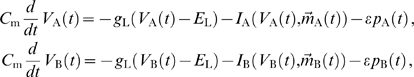
(3)where 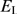 is the reversal potential of the passive membrane current, the function 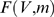 summarizes the voltage-dependent terms in the cable, 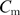 is the membrane capacitance, 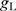 is the leak conductance, 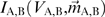 describes the voltage-dependent currents generating the oscillations. The gating variable 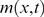 and the variables in the vector 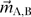 are described by standard kinetic equations (e.g. see Equations 28 and 29 in Methods). The terms 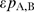 describe the perturbing currents that each oscillator receives from the cable and are proportional to 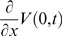 and 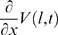. A more detailed description for the above is given in the Methods.

The two oscillators described by Equation 3 form the boundary conditions Equation 2 for the cable Equation 1. In turn, the cable yields the current flux through its ends into (and thereby perturbing) the two oscillators: the terms 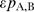 in Equation 3. It is clear that it is next to impossible to solve Equations 1–3 directly. However, we will use a number of reductions to arrive at a phase description of the system that is simple enough to handle analytically. This allows us to derive interaction functions for the two oscillators, describing how much they perturb each other through the dendrite depending on their phases. We then use these interaction functions to determine the stable phase relationship between the oscillators for different parameters, i.e. the properties of the cable and the type of oscillators. The analysis follows along the lines of previous work [26]–[28] and extends those results to the analysis of intradendritically coupled oscillators.

We begin by observing that the oscillators from Equation 3 can be reduced to a phase description (see Methods for further detail) [24]. The phases 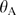 and 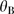 (in radians) describe the state of each oscillator. The dynamics of the phases are then described by 
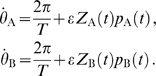
(4)Here the first term in the right hand side of each equation is the natural frequency of each oscillator and the second term describes the interaction between the oscillators. The crux of the analysis is thus to derive this function which we do explicitly in Methods.

The interaction between the two oscillators depends on two factors: the intrinsic properties of the oscillators, as reflected by their phase response curves 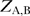, and the perturbations 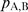 to each oscillator via the cable. A phase response curve of an oscillator describes the phase shift induced by a perturbation delivered at a given phase. It can be determined using standard methods [24]. The perturbations to the oscillators come from solving Equation 1 with the oscillators described by Equation 3 as the boundary conditions described by Equation 2. For the active cable this task can be greatly simplified if we consider a quasi-active approximation of the cable, and if we realize that the cable should behave periodically. The former can be done by linearizing the cable Equation 1 about the voltage to which the cable would relax if it was not driven by the oscillators [29],[30]. Under such approximations the active properties of the dendritic cable can be summarized by a single parameter, 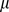, which can be derived from its basic biophysical properties (see Methods). The sign of 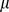 indicates whether the active conductance that is present in the cable is regenerative (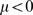), restorative (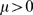), or passive (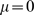) (see also [28]). A regenerative current will amplify perturbations (e.g. a persistent sodium current 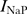), while a restorative current actively counteracts such perturbations (e.g. the hyperpolarization activated inward current 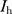).

Since the solution to the cable equation with periodically forced end conditions is also periodic, it depends only on the difference of the phases of the two oscillators 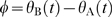. The dynamics of 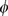 is the central object of our interest. Assuming that the oscillator interactions via the cable are relatively weak, we can obtain the interaction functions 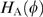 and 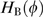 (see [24] and Methods). These describe the change in the oscillators' phases as a function of the phase difference. Now the phase difference between the oscillators evolves, on a slower time-scale, as 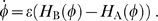
(5)


It is easy to see that phase-locked states for our dendrite can be identified as values of 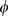 where 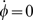. The derivative of 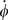 with respect to 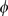 gives the stability of such states (negative implies stable, positive unstable). Hence for the rest of the analysis we study how stable phase-locked configurations are determined by the key biophysical parameters of the system described by Equations 1–3: the electrotonic length and membrane time constant of the cable, the nature of the active cable-currents, the frequency of the oscillators, as well as the properties of the oscillators as given by the phase response curves and the voltage trajectory shape.

### Phase-locking with simplified dendritic oscillators

The basic behavior of the system can be most easily understood by examining a simplified situation where the oscillators have a phase response curve that is approximately sinusoid and the perturbations from the cable are also nearly sinusoidal (e.g. when the oscillators are subthreshold with simple sinusoidal voltage traces). Hence the first Fourier component dominates in both 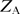 and 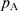. The interaction function is then 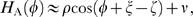
(6)where 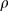 is a positive coefficient characterizing the strength of the coupling (see Equation 22 in Methods). The term 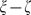 gives the effective phase delay in the interaction between the two oscillators (figure 1Aii). In this term 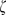 depends on the properties of the oscillators and 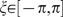 summarizes the effect of cable filtering. It depends on the properties of the dendritic cable: 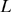, 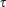, and 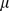 (see Methods). Using Equation 5 it is easy to show that the evolution of the phase difference 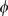 between two identical oscillators is given by 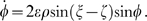
(7)


The fixed points are the in-phase solution 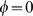 and the anti-phase solution 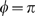 (figure 1Aiii). The stable phase-locked solutions are those fixed points where the derivative of Equation 7 with respect to 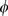 is negative: 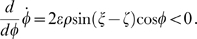
(8)


The synchronous solution 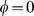 is thus stable when 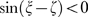. When this solution is stable the anti-phase solution 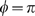 is unstable and vice versa.

Notice that if we fix the properties of the oscillators, the constant 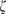 is fixed. Then the value of 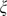 uniquely determines which is the stable state (figure 1Aiv). Hence, to understand how the dendrite behaves as a function of the key properties of the cable we need only to look at how these affect 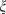. In the next sections we describe the behavior of 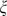 with the consequent effect on phase-locking. The explicit expressions for the scaling of 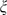 with the various parameters considered below are given in the Methods.

#### Passive cable properties and oscillator period set the phase-locked states

First let us consider a setup where the cable is passive (i.e. 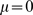). We show how 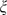 depends on the various cable parameters as well as the oscillator period and by extension how these properties affect the phase-locking.

 The electrotonic distance 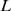 between the oscillators is one of the major determinants of 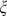. For a fixed membrane time constant and oscillator period, the electrotonic distance controls the amplitude of 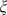. For example, let us set the membrane time constant 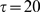 ms and the oscillator period 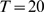 ms. As we let 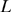 increase from 0 to 8, 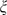 moves through almost two whole cycles (figure 1B). Thus, the in-phase and the anti-phase states exchange stability as a function of 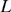. There are ranges of 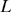 where 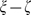 is negative so the right hand side of Equation 8 is below zero and the in-phase solution is stable (white area in figure 1B), and ranges where 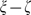 is positive and the anti-phase solution is stable (grey areas in figure 1B). Hence for different electrotonic lengths we observe either coherent synchronous or anti-phase voltage oscillations. Our analysis also shows that, for large enough 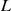, the transitions between the stability of in-phase and anti-phase solutions are periodic with respect to 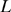 (see Equation 26 in Methods). The period 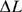 of these transitions depends on the cable time constant 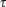: e.g. for increasing 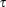 the transitions between the phase-locked modes come at shorter cable lengths. Note that we vary the electrotonic distance 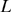 here over a large range in order to highlight the periodicity of the transitions. A more physiologically realistic limit on the maximal 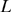 that is attainable within a neuron is on the order of 4 length constants [31].

Thus we see that the spacing of the oscillators can determine if they would produce global synchronous oscillations or not. Interestingly, the relationship between the spacing and synchrony is not trivial since synchrony can result both at short and long electrotonic distance. The electrotonic distance can be influenced by the conductance state of the cable, hinting that the level of synaptic input impinging on the cable may determine the phase-locked states in a non-trivial manner. To examine this issue explicitly we look at the relationship between 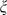 and the membrane resistance 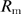 of the cable.

Both the membrane time constant 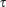 and the electrotonic length 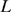 of the cable depend on 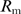. In a low conductance state, as 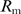 grows large, 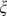 approaches a constant. So the influence of 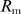 on 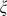 and hence the phase-locked state saturates. For example in figure 1C, only the anti-phase solution is stable for large 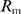. On the other hand, in a high conductance state of the dendrite 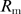 becomes small, driving 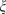 towards zero. In this range 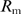 has a strong effect on 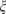 and can therefore change the stable phase-locked solution. For example, see in figure 1C when 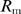 is below 10 k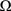 cm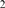 (corresponding to a membrane time constant of 10 ms).

So far we have shown how basic properties of the cable connecting the oscillators determine the phase-locking regimes. However, the period 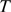 of the oscillators also plays an important role in setting the phase-locking by affecting the amplitude and sign of 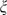. In figure 1D we plot 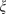 as a function of the oscillation frequency (in Hz) with an electrotonic distance between the oscillators of 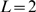 with 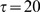 ms. We can see that by changing the frequency of the oscillators one can change the stable phase-locked solution from in-phase to anti-phase or vice versa as the value of 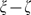 changes sign (i.e. as 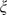 moves from the white to the grey areas or vice versa in figure 1D).

Hence the stability of the phase-locked solutions can be determined by basic properties of the cable, such as the electrotonic distance and/or the membrane resistance, as well as the properties of the oscillators, such as their frequency. Next we see how active properties of the cable can set the phase-locking regimes.

#### Active cable properties influence phase-locking regimes

Voltage-dependent ionic conductances in the dendritic cable that connects the oscillators strongly modulate 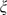. Let us consider phase-locking as a function of 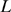 for the various active cable currents, such as 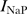 (regenerative) and 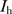 (restorative).

**Figure 2:**
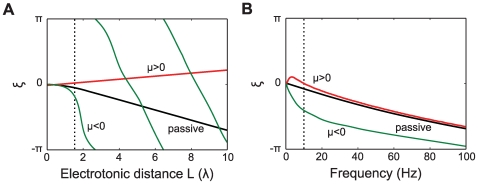
Active cable coupling. A: Parameter 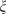 as a function of the electrotonic distance 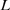 between the oscillators when the cable is passive (black) or with a regenerative (green) or a restorative (red) active current. The oscillator frequency is 8 Hz (dotted line in panel B). The membrane time constant of the connecting dendrite is 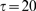 ms. The parameters for the active currents were determined for 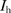 (restorative) and 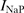 (regenerative) which are described in the Methods (see Equation 29). The current parameters when linearized around 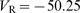 mV are 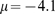, 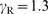 and 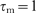 ms for the regenerative current, and 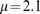, 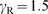 and 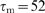 ms for the restorative current, using the conductance densities given in the Methods. B: 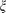 as a function of the frequency of the oscillator (in Hz). The oscillators are separated by a cable with electrotonic length 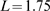 (dotted line in panel A) for the same three conditions as in panel A.

Regenerative currents (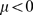) make 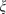 more sensitive to 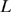, causing transitions of stability to occur on shorter intervals 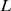 as compared to an equivalent passive case. This is illustrated in figure 2A: with a regenerative current (green curve) 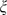 goes through more than two complete cycles as 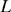 increases from 0 to 10. For the passive cable case (black curve) there is a shift of only about a third of a cycle for the same range of 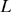. In contrast, restorative currents (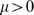) typically have the opposite effect, making the intervals 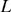 between the transitions longer. For example, in figure 2A one can see that the restorative current (red curve) leads to a small increase in 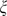 with increasing 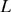 and effectively removes the effect of the electrotonic distance on 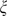.

The way active currents modulate the relationship between 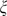 and 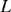 also depends on the frequency of the oscillators. In panel 2B we plot the frequency-dependence of the 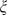 for the regenerative, restorative and passive cable currents when 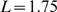. The restorative current yields a positive value of 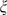 up to a frequency of 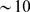 Hz. The regenerative current increases 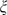 compared to the passive cable most strongly for low frequencies. For both restorative and regenerative currents the effects on 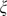 disappear for very high frequencies.

### Phase-locking dynamics of multiple complex oscillators

In the previous section we limited our description and analysis to oscillators with a nearly sinusoidal phase response curve that receive perturbations which are also sinusoidal. In this way we could demonstrate how the parameters that define the oscillator and cable properties affect the phase-locking behavior of the system. However, as consequence, we only obtained and analyzed symmetric interaction functions 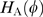. For such coupling functions, only the in-phase and anti-phase solutions are possible of which one is stable and one unstable. When 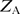 and 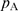 cannot be well approximated by a single Fourier component we need to take into account higher order terms. Including more Fourier components is likely to lead to asymmetry or skew of 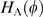 and, as we will show next, this affects the possible phase-locking behaviors.

#### Skew of the interaction function determines the possible phase-locked states

We will now consider how the skew of the interaction function 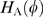 affects the phase-locking behavior. To illustrate this point let us look at a sawtooth-shaped 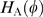 with period 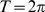 that increases from 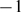 to 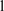 over the interval 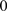 to 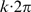 and decreases back to 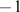 on the remaining interval. The parameter 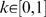 thus specifies the location of the peak such that for 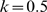 we have a standard triangle wave. We assume identical oscillators such that 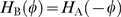. For illustrative purposes we first consider a somewhat artificial yet illustrative example, in which the cable filtering does not affect the shape of the interaction function but only shifts the interaction function along the 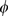-axis. We define a single parameter 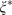 that determines the position of the interaction function 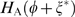, analogous to 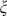 in the above analysis. This parameter 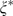 depends on the various parameters in a way similar to 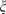, for example with the electrotonic separation of the oscillators.

**Figure 3:**
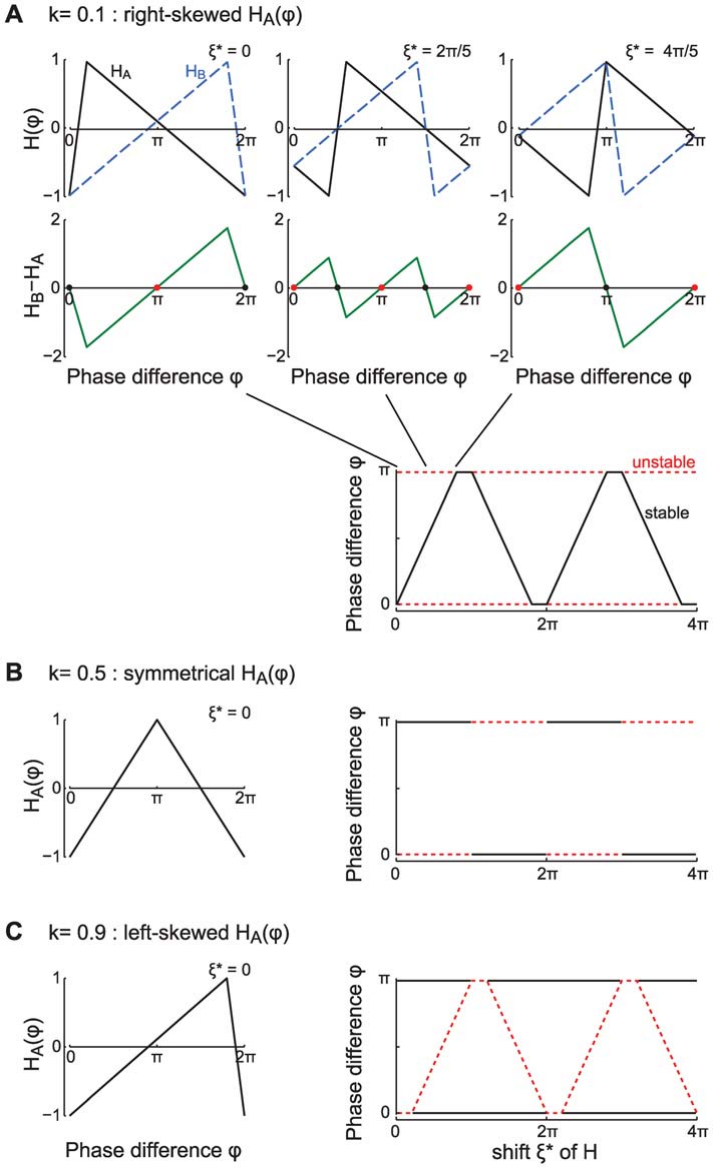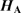 skewness controls phase-locking regimes and transitions. The three panels A-B-C show triangular 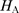 functions with different skewness with their peaks at 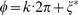 where 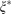 is a phase shift that results from the cable coupling. The oscillators are identical so that 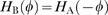. A: Right-skewed 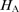 with 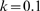 (solid black line) plotted from left to right for three values of 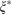 together with the corresponding 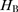 (dashed blue line). Below each graph 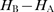 is plotted (green lines) with the stable (black dots) and unstable (red dots) phase-locked solutions. The lower right panel shows the bifurcation diagram with the stable (solid black line) and unstable (dotted red line) phase-locked solutions. The right-skewed 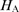 yields gradual transitions between the in-phase and anti-phase solutions. B: Symmetrical 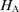 with 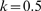 yields abrupt transitions between in-phase and anti-phase solutions. C: Left-skewed 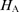 with 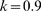 yields bistable regions where both the in-phase and the anti-phase solution are stable.

The skew of 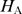 leads to a richer repertoire of phase-locking which we demonstrate in figure 3. We first consider a right-skewed 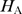 with 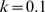. The top panels in figure 3A show 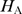 and 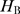 for three different values of 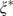. Below these panels we plot the difference 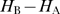 from which we can read the phase-locked solutions since these are given by 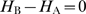 (see Equation 5). We see that the interaction functions 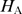 and 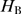 move in opposite directions along the 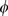-axis as 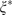 varies from 0 to 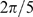 to 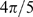. The bifurcation diagram in figure 3A (lower panel) shows the stable and unstable phase-locked solutions as a function of 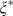. Hence we see that not only in-phase and anti-phase solutions are possible, but also phase-locked solutions at intermediate values of 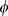. Thus, a right-skewed 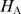 (i.e. when 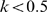) leads to gradual transitions between in-phase and anti-phase solutions. As we noted above, when 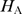 is symmetrical (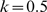) we find only instantaneous transitions between in-phase and anti-phase solutions (figure 3B). Finally, for a left-skewed 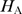 (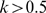) one finds parameter ranges with simultaneous stability of both the in-phase and the anti-phase solution (figure 3C).

#### Factors determining the shape of the interaction function

The actual shape of 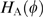, and consequently the bifurcation diagram governing the dendritic phase-locking, depends on the properties of the oscillators and the cable. If we know the voltage trace and phase response function of an oscillator, we can easily compute the interaction function for direct coupling using Equation 27 in the Methods. The skew of the interaction function then predicts the type of phase-locking behaviors that can be expected. For spiking oscillators one will typically find a left-skewed voltage trace as the membrane potential gradually approaches the threshold and the spike is followed by a quick reset. For such an oscillator, a symmetric phase response function will yield a left-skewed interaction function and one expects to find bistable phase-locking regimes. For subthreshold oscillators, the voltage trajectory is more likely to be symmetric. The skew of the phase response function will then determine the skew of 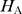.

However, when we introduce an electrotonic separation 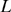 between the oscillators, the shape of the interaction function 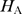 will change as a result of the cable filtering. As 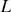 increases, the increasing cable filtering leads to dominance of a single Fourier component. Thus, for large 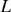 the shape of the interaction function will always approach that of a sinusoid. As a consequence one expects to see abrupt transitions between the phase-locked solutions as 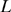 becomes large. See also the “Skew of interaction function” section in the Methods.

#### Behavior of specific oscillator models

As we mentioned above, the shape of the interaction function depends critically on the biophysics of the oscillators considered. Hence, we now turn to illustrating our analysis for two different oscillator types: one that generates action potentials and the other a model for subthreshold oscillations.

**Figure 4:**
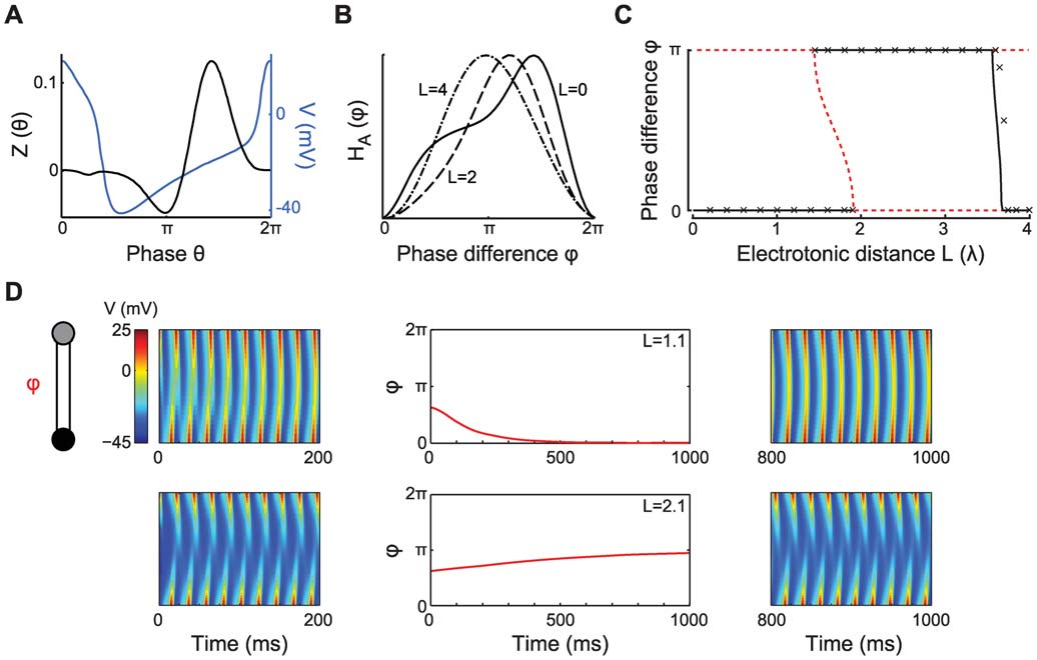
Phase-locking of two Morris-Lecar type II oscillators. The oscillators (described in Methods) are coupled via a passive cable of electrotonic length 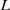, 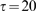 ms. A: Voltage trajectory (blue) and phase response function (black) of the Morris-Lecar type II oscillator, period 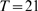 ms. B: Shape of 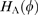 for 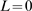 (solid curve), 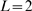 (dashed curve) and 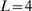 (dash-dotted curve). The functions have been rescaled and aligned in order to show the different degrees of skewness. C: Bifurcation diagram showing the stable (solid black line) and unstable (dashed red line) phase-locked solutions as a function of 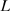. Cross marks give the stable phase difference determined with numerical simulations using 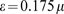
S cm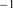 with 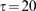 ms, and 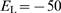 mV. D: The middle two panels show simulations of the phase difference dynamics (red curves) for 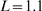 (top) and 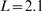 (bottom) with 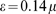
S cm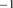. Space-time plots of the membrane potential along the dendritic cable cable are plotted for the first 200 ms (left) and for the final 200 ms (right) of the two simulations.

As a first example we analyze the phase-locking for the type II Morris-Lecar neural oscillator [32] (see Methods). We also validate our analysis with direct numerical simulations. We first focus on the relationship between 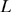 and the shape of 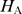 for this oscillator type. The voltage trace and the phase response function of this oscillator are plotted in figure 4A for one oscillation cycle, starting at the peak of the voltage trace. The interaction function 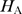 is shown in figure 4B for three values of 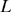. For 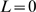 we have two directly coupled Morris-Lecar oscillators, resulting in a left-skewed 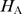 (solid curve). For 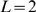 (dashed curve) the interaction function has become smoother, though it is still left-skewed. For 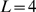 (dash-dotted curve), most high frequency components are filtered out as a result of the cable filtering, and we have an almost symmetric 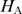. From this we expect that if there is a transition between stability of the in-phase solution and stability of the anti-phase solution for 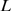 smaller than 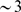, that this transition will be accompanied by a bistable region surrounding that transition. For larger 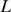 the transition will be practically instantaneous. This is indeed what we see in the bifurcation diagram in figure 4C, which shows the stable (black) and unstable (red) phase-locked solutions as a function of the electrotonic distance 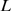. As expected for a left-skewed 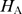, the dendrite shows a bistable region where both the in-phase and the anti-phase solution are stable (around 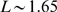). For smaller 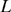, the in-phase solution is stable. As the electrotonic separation between the oscillators approaches 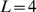, there is also a transition from a stable anti-phase to a stable in-phase solution. This transition is very sharp, as was expected for the almost symmetric shape of 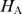 at this electrotonic distance.

Using numerical simulations of Equations 1–3 (see Methods) we can demonstrate the dynamics of the phase difference between the two Morris-Lecar oscillators, as well as the membrane potential dynamics along the cable. Figure 4D illustrates these dynamics when the oscillators are separated by an electrotonic distance of 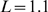 (top panels) or 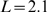 (bottom panels). The oscillators start out with a phase difference of 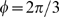. As expected from the bifurcation diagram in figure 4C, the two oscillators move to the in-phase configuration 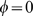 when 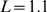, synchronizing the voltage oscillations along the cable. When 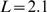 the two oscillators settle in the anti-phase solution 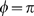, producing large voltage gradients along the cable.

**Figure 5:**
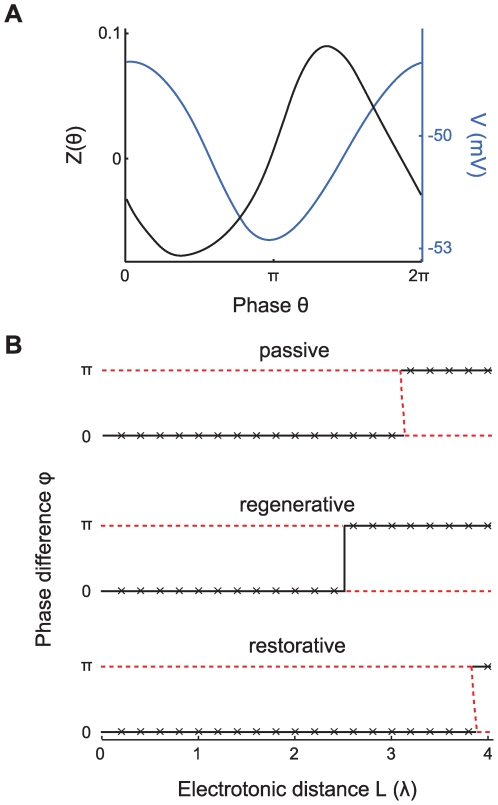
Phase-locking behavior of subthreshold oscillators. The oscillations are generated by interactions between 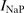 and 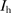 (see Methods). A: Voltage trajectory (blue) and phase response function (black) of the oscillator. B: Corresponding bifurcation diagrams showing the stable (solid black lines) and unstable (dashed red lines) phase-locked solutions as a function of 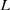. The bifurcation diagram is shown for a passive cable (top), a cable with a regenerative current (middle), and a cable with a restorative current (bottom). The restorative current 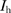 and regenerative current 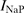 (described in Methods) are inserted in the cable with relative densities of 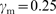 and 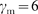, respectively. Linearizing these currents around 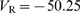 mV gives the parameters 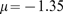, 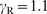 and 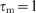 ms for the regenerative current, and 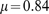, 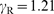 and 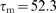 ms for the restorative current. The membrane time constant of the connecting dendrite is 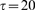 ms. Cross marks in the bifurcation diagrams give the stable phase difference determined with numerical simulations using 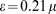
S cm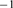, 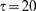 ms, and 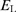 is 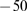 mV, 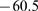 mV and 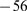 mV, respectively for the three panels, so that the cable's resting potential is 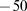 mV. Note that the numerical simulations use the original (i.e. not the linearized) active currents in the connecting cable.

Finally, we determine the phase-locking under both passive and active cable coupling for a model of subthreshold oscillations in entorhinal stellate cells [6],[33] (see Methods). These oscillations are thought to arise from an interaction between a persistent sodium current 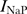 and a hyperpolarization-activated inward current 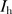 (see Methods). Both the voltage trajectory and the phase response function are close to a sinusoid (figure 5A). We compute the bifurcation diagrams (figure 5B) for two oscillators coupled via a passive cable (top), a cable with a regenerative current (middle), and a cable with a restorative current (bottom). As was expected from our above analysis for simplified oscillators, the regenerative current makes the transition between in-phase and anti-phase solutions to occur for smaller 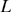, compared to passive cable coupling. In contrast, adding the restorative current to the cable causes the transition to occur at larger 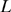, making the synchronous phase-locked solution stable up to 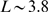.

#### Numerical simulations agree with predictions of weak coupling analysis

Our mathematical analysis assumes that the oscillators are weakly perturbed by the coupling via the dendritic cable. This implies that the currents in the stretch of cell membrane that generate the intrinsic oscillations are much stronger than the perturbing currents that arrive from the dendritic cable. Hence, central parameters determining the coupling are the amplitude of the oscillator's intrinsic currents and the parameter 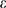 in Equation 3, which should be such that the ratio of the amplitudes of the perturbing current and the intrinsic currents 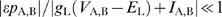. For a cable with diameter 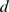 (in cm) and oscillators that are described as a single isopotential compartment with membrane surface area 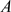 (in cm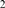), the parameter 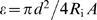, where 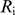 is the intracellular resistivity of the dendritic cable (in k
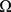
cm). The analytical prediction of the stable phase-locked state will become less accurate as 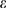 grows, for example when the oscillator's length and hence its surface area become smaller.

Using numerical simulations of Equations 1–3 (see Methods) we tested how well the weak coupling approximation predicts the phase-locking of the oscillators, both for the type II Morris-Lecar oscillators (figure 4) and the subthreshold oscillators (figure 5) when coupled via a cable with an electrotonic length ranging from 0 to 4 length constants, with membrane time constant 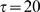 ms. We find that the analytical predictions agree very well (cross marks in figure 4C and figure 5B) when we use up to the maximal 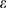 that still allows for oscillations (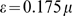
S cm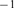 for the Morris-Lecar oscillators and 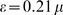
S cm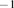 for the subthreshold oscillators). Larger values of 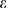 lead to such strong interaction currents that the oscillations are annihilated. Numerical simulations of Equations 1–3 using voltage-dependent cable currents (see Methods) match exactly with the predictions of the weak coupling analysis (bottom two panels in figure 5B), thereby also emphasizing the validity of using linearized descriptions of those active currents in our analytical framework.

Finally, we also simulated a cable in which we inserted the voltage-dependent conductances that underlie the Morris-Lecar type II oscillator in the end segments (see text S1 in Supporting information). Hence, this continuous cable model does not use the explicit assumption of weak coupling. Results from these simulations also agree with our analytical predictions, showing synchronized phase-locking for small 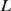, a bistable regime around 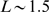 and anti-phase locking for larger 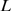 (see text S1 and figure S1 in Supporting information).

#### Multiple oscillators: chains and branched structures

**Figure 6:**
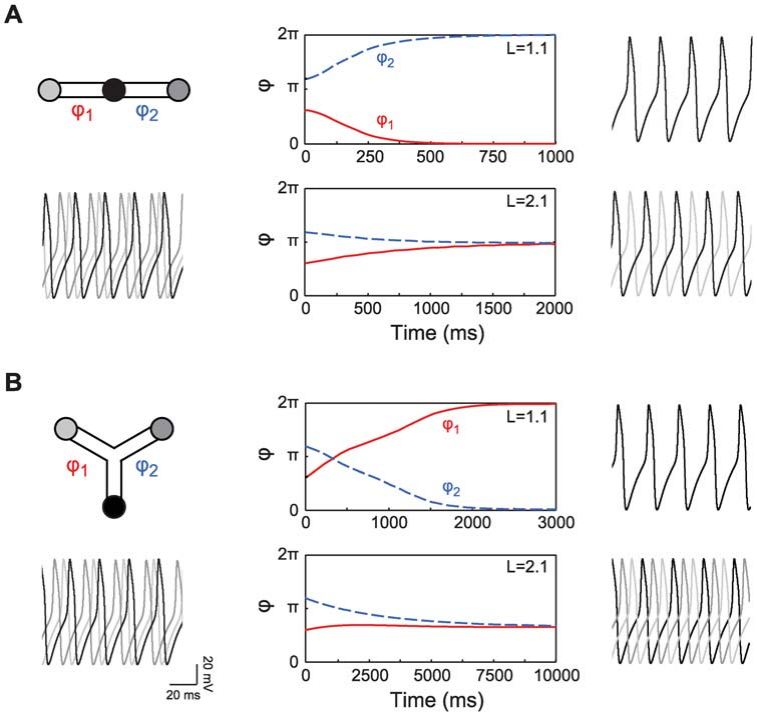
Phase difference dynamics of three oscillators in a chain or a branched configuration. The Morris-Lecar type II oscillators are separated by a passive cable, 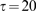 ms. Panels A and B show from left to right: a scheme of the model with below it the membrane potential of the oscillators at the start of the simulation; the dynamics of the phase difference 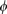 between the oscillators for 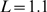 (top) and 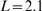 (bottom); and the membrane potential of the oscillators at the end of the simulation. The properties of the Morris-Lecar oscillators and the dendritic cable are as in figure 4.

So far we have focused on a minimal configuration of two oscillators connected by a cable. However, our analysis can be easily extended to predict phase-locking of a chain of oscillators. This follows since the phase-locking behavior only depends on each neighboring pair of oscillators. Figure 6A shows numerical simulations of a chain of three oscillators, using the same Morris-Lecar model as in figure 4. The two pairs are separated by a passive dendritic cable of either 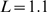 (top panel) or 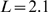 (bottom panel). The phase-locked solutions follow from the bifurcation diagram in figure 4C: the three oscillators move into an in-phase solution for 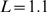, whereas for 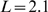 each neighboring pair of oscillators moves into the anti-phase solution.

Our framework also allows us to understand phase-locking in a branched cable structure. Hence we examined the phase difference dynamics of a triangular configuration of three Morris-Lecar oscillators (figure 6B). In this situation, each oscillator is separated from the other two oscillators by a passive dendritic cable with electrotonic length 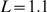 (top panel) or 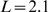 (bottom panel). For 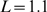, all three oscillators synchronize. When 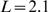, we expect from the bifurcation diagram in figure 4C that the oscillators go into anti-phase. However, as we have three mutually coupled oscillators, two pairs of anti-phase locked oscillators would lead to an in-phase configuration of the the final pair of oscillators. The bifurcation diagram shows that the in-phase configuration is unstable. We see from the simulation that the system settles into the solution closest to the anti-phase solution, which is a phase difference of 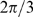 between each pair of oscillators.

### Dendritic phase-locked states: controlled by inputs and read out with spikes

**Figure 7:**
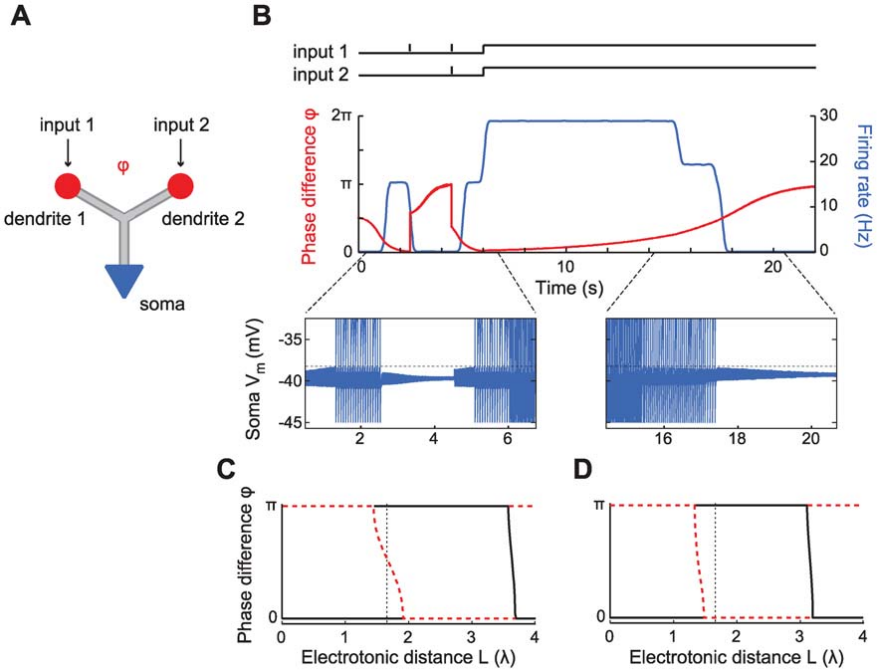
Changing the phase-locked solution of dendritic oscillators with external input and its detection with an excitable soma. A: Schematic drawing showing the configuration of two dendritic Morris-Lecar type II oscillators and a spike-generating soma (see Methods). All are separated by a passive cable with electrotonic length 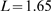 and 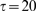 ms, with 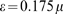
S cm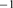. B: From top to bottom are shown the inputs to the two dendritic oscillators, the phase difference dynamics (red) and somatic firing rate (black), and the somatic membrane potential 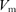 (blue) with the spike threshold (dotted black line). Note that the spikes have been cut off in order to show the subthreshold membrane potential. C–D: Bifurcation diagrams describing the phase-locked solutions up to 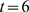 seconds (C, see also figure 4C) and after 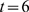 seconds (D) with dotted line at 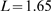 giving the electrotonic distance between the dendritic oscillators.

Above we developed a framework for analyzing the behavior of local oscillators embedded in the dendritic tree. Now we turn to the question of how such oscillating dendrites respond to inputs and impact the output of the neuron. We will show that the external synaptic input can control the phase-locked configuration of the dendritic oscillators and that this phase-locked configuration can then be transmitted through patterning of the cell's action potentials. While a thorough analysis is beyond the scope of the present study, we give several salient illustrative examples using a model with a branched oscillating dendritic tree and a spike-generating soma. More specifically the model consists of a passive branching dendritic compartment with two Morris-Lecar type II oscillators at its two distal ends and an excitable soma that, for simplicity, we describe with an integrate and fire mechanism (figure 7A).

Above we showed that under certain conditions, depending on the skew of the interaction function 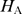, the dendritic tree can be in a phase-locking regime where two stable phase-locked states co-exist (see figures 3C and 4C). In such a bistable regime, well-timed inputs to one or more dendritic oscillators can switch the locking between in-phase and anti-phase. Clearly, the membrane potential fluctuations at the soma depend on whether the dendritic oscillators are synchronized or not. In our model, they are largest in amplitude when the dendritic oscillators are in-phase. The soma can show this difference with its spiking pattern when such large amplitude fluctuations are supra-threshold, while smaller fluctuations (e.g. with asynchronous oscillators) are not.

In figure 7 we illustrate the above mechanism. The initial parameters are such that both the in-phase and anti-phase state of the dendritic oscillators are stable (black dotted line in figure 7C). Oscillators starting from an initial phase difference 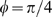 move into the synchronous phase-locked state (red curve in figure 7B). This consequently leads to repetitive somatic spiking (blue traces in middle and bottom panel). A brief depolarizing current pulse to one of the oscillators (see black trace in top panel of figure 7B) moves them into the anti-synchronous state and the somatic spiking ceases. A subsequent synchronous current pulse to both dendritic oscillators can switch them back into the synchronous state and hence restart the spiking. Note that all the stimuli here are excitatory, yet depending on their timing, they can have a net excitatory or inhibitory effects on the cell's spiking.

We have also hinted, in a previous section, at another mechanism by which inputs to the dendrites can affect the phase-locked state. The input amplitude can change the oscillator frequency which in turn has an effect on the stability of the phase-locked state (see figure 1D). In figure 7B at time 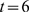 sec we increase the amplitude of the current input impinging on the oscillators which causes the system to move out of the bistable regime. The synchronized state loses stability and the oscillators gradually move into anti-phase locking. As a result, the soma stops spiking (at time 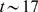 sec). Note that the electrotonic separation between the oscillators remains constant (black dotted line in figure 7D) but that the bifurcation diagram itself changes. In turn, a decrease in the excitatory input would reinstate spiking. Hence, this mechanism allows the cell to encode an inverse of the input amplitude, or the inverse of the excitatory input rate.

## Discussion

The question of how local cellular processes may lead to global behavior has been of great interest for some time, in particular with respect to the signal propagation in extended structures such as the dendritic trees of cortical neurons. One of the aspects that remains a subject of active debate, is the dendritic mechanisms that ensure that local inputs on the dendrites – and in particular on the distal dendrites – have an impact on the global signal processing in the cell and ultimately on spike generation. We addressed this key question focusing specifically on the case of oscillatory dendrites. Thus, we studied the dynamics of dendrites that show intrinsic oscillations due to active voltage-dependent currents that present strong spatial inhomogeneities, hence leading to discrete oscillatory segments. Our prime question was to understand how global dendritic behavior, in this case the phase-locked oscillations, can arise from interactions between such local oscillators. To do so we developed an analytical framework to describe and understand the behavior of interacting dendritic oscillators and their impact on signal propagation within a neuron. Our goal was to understand when the oscillators within the dendrite would lock and hence the whole dendritic tree would act as a single oscillatory unit.

 Using the weakly coupled oscillator framework we have identified the requirements for the various phase-locking regimes of the dendritic oscillators. We characterized how the type of phase-locking depends on the intrinsic properties of the oscillators as well as on the membrane properties of the dendrite segment connecting them. We find that a central parameter in determining the phase-locked solutions is the electrotonic distance between the oscillators. This distance determines how strongly the dendritic cable filters the interactions between the oscillators, thereby determining the delay between the interactions. As a function of the electrotonic distance the phase-locking of identical oscillators alternates between in-phase or synchronized solutions and anti-phase solutions.

We also showed how the phase-locking is affected by the presence of voltage-dependent conductances in the cable that connects the oscillators. Using the quasi-active approximation of the cable [29],[30] we found that the dependence of the stable phase-locked solution on the electrotonic distance is typically amplified by regenerative conductances (i.e. ionic conductances that amplify a voltage perturbation), whereas it is counteracted by restorative conductances (i.e. ionic conductances that counteract voltage perturbations) (see also [28]). It should be noted that the linearization of the active conductances in the dendrites is appropriate for small amplitude oscillations in the dendrite and is therefore in general a better approximation for subthreshold oscillations than for spiking oscillators.

The mathematical approach that we used, builds on several studies which focused on the interaction between two neurons with repetitively spiking somata that interact via inputs at the dendrites [26]–[28]. A crucial difference with these studies is that rather than coupling via discrete synaptic events, we treat continuous coupling between the oscillators via the current-conducting cables. One consequence of the continuous coupling is that one needs both the phase response function and the voltage trajectory of the oscillators in order to compute the interaction functions and ultimately the phase-locked solutions. By computing the convolution of the voltage trajectory and the phase response function, which yields the interaction function for directly coupled oscillators, it is possible to get some insight into the types of phase-locked solutions that can be expected. The skew of the interaction function can show whether regimes can be expected in which both in-phase and anti-phase solutions are stable. Both the voltage trajectory of an oscillator and its phase response function can be determined numerically from a model of an oscillator and, at least in principle, also experimentally (see, for example, [34]).

In the final section of our study we demonstrated how inputs to the dendritic tree can set the phase-locked state and how in turn the phase-locked configuration can control somatic spike generation. The first can for instance be accomplished by changing the frequency of the oscillators with the external input. The soma can subsequently detect the amplitude of the membrane potential fluctuations since this is affected by the phase-locked configuration. The time scale at which the dendritic oscillators move from one solution to another is set by the strength of the interactions between the oscillators. This time scale can be much longer than that of the different components of the system, e.g. the membrane time constant or the period of the oscillators. In this way, the phase difference between the oscillators can function as a memory. Related ideas have been previously discussed by Huhn et al [35]. We also showed that in the bistable phase-locked regime the state of the dendrites is easily set by transient inputs and “read-out” by the soma. This also can endow the neuron with a memory since brief external inputs can switch the neuron from a spiking to a quiescent mode and vice versa. Interestingly we showed that both the turn-on and turn-off signals (inputs) can be excitatory, their final effects defined by their timing.

The focus of our report is complementary to that of a recent theoretical study of the subthreshold oscillations in the dendrites of mesencephalic dopaminergic neurons [36]. As these cells do not show any indication of distinct dendritic oscillators, the whole cell was modeled as one continuous oscillator with gradients in oscillator properties along the dendrites. Moreover, since there were no distinct oscillators, in their analysis Medvedev and colleagues assumed strong voltage coupling between neighboring compartments, enforcing synchronized oscillations throughout the cell. In contrast, our approach assumed weak coupling between the dendritic oscillators. This would not be appropriate for a spatially continuous oscillator. However, it is not possible to state in general at what precise electrotonic distance between two oscillators the weak coupling assumption becomes valid, since it depends on the strength of the interaction currents with respect to the intrinsic currents of the oscillators. However, our numerical simulations for a dendritic cable without the assumption of weak coupling, show that the phase-locking behavior of Morris-Lecar oscillators is consistent with weak coupling.

One of the aims of the present paper was to set up an analytical framework for studying interacting dendritic oscillators. This opens up a wide range of questions that were outside the scope of the present study. For example, we focused our analysis on identical oscillators, while it is likely that dendritic oscillators will vary in their properties throughout the dendritic tree. For example, the diameter of the dendrites, which typically becomes smaller with increasing distance from the soma, can affect the intrinsic frequency of the oscillators. A gradient in the frequency of distinct oscillators is likely to lead to more complex phenomena such as traveling waves (see, for example, [37]).

 In fact the major focus of our study is to explore how local dendritic mechanisms may lead to oscillations expressed globally in the cell and hence visible at the soma, for example in somatic intracellular recordings. Our analysis showed that even electrotonically far removed dendritic oscillators can lead to voltage oscillations that significantly affect the soma voltage and hence spike generation. This suggests several experimentally testable predictions. In one possible experiment one can take advantage of imperfect space clamp in a electrotonically extended neuron. As a proof of principle, in a neuron where the oscillations are generated distally in the dendritic tree, voltage clamping the soma would not block such oscillations, and these should be seen in the current necessary to hold the somatic potential. In fact, results from [18] point in this direction, where in chick spinal cord neuron NMDA-dependent intrinsic oscillations were not blocked by somatic voltage clamp. A further prediction stems from the weak coupling between active dendrites. If active oscillations, such as periodically generated dendritic spikes, are generated in different segments of the dendritic tree, our analysis predicts that such spikes should interact and should exist in a stable phase-locked configuration, e.g. synchrony. Hence, should one of the dendritic segments be phase-shifted, such perturbation should 1. propagate to the other segment (the other segment should be phase reset) 2. the dendritic spikes should return to the phase-locked configuration 3. the time scale of this return should be relatively long and determined by the electrotonic distance between the active segments. While difficult such experiments are possible using the multiple dendritic recording techniques, such as those developed by Davie et al [38] in Purkinje cells.

A recent model for the grid field properties of the entorhinal cortex layer II stellate cells [21],[22],[39] relies precisely on the ingredients considered in the present study. The model assumes that different dendritic branches emanating from the soma of these cells function as distinct oscillators. The oscillations are modulated by external inputs and the interference of the oscillators eventually determines the somatic spiking. Crucially, the model assumes that the dendritic oscillators operate independently. At a first glance, our results appear to argue against this: the various oscillators should phase-lock (hence lose their independence) even when the mutual coupling is weak. However, in principle, the locking may be slower than the behavioral time scale, allowing the oscillators to act quasi-independently on the behavioral time scale. Our analysis provides the appropriate framework to examine these issues: the scaling of locking in time and the biophysical implementation of grid-field formation via dendritic oscillators.

Above we studied relatively simple cell geometries, however these form basic building blocks for more complex dendritic trees. Thus our framework should be valid for understanding global voltage oscillations in more realistic models of spatially extended cells. We would like to emphasize at this point that our general framework should also hold when – in addition to the distinct oscillators distributed throughout the dendritic tree – also the soma is regarded as an oscillator. These and other issues will be addressed in future publications.

The framework we have developed, builds on the extensive mathematical theory of coupled oscillators and nestles nicely below the complexity of full compartmental models of neuronal dendritic trees. Yet our framework is sufficiently powerful and clear to both take into account certain key aspects of the dendritic tree structure and to be amenable to theoretical analysis of the dynamics of active dendrites and the computational function of such dendritic structures. These remain an active focus for further investigations.

## Methods

### Interaction functions for two weakly coupled dendritic oscillators

We analyze the behavior of a system of two oscillators that are coupled via a cable. For this we need to compute the interaction between the two oscillators. Our approach is as follows. The oscillators provide the periodically forced end conditions for the cable equation. Assuming weak coupling the phase difference between the oscillators does not change significantly within one period of the oscillation. Thus we can solve the cable equation with such boundary conditions and leave the phase difference as a free parameter. In turn, the solution of the cable equation yields the currents flowing into and thereby perturbing the two oscillators at its ends.

We let 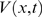 denote the membrane potential (in millivolts) along the cable at position 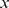 (in centimeters) and at time 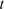 (in milliseconds). The passive properties of the cable are determined by a membrane time constant 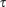 (in milliseconds) and a length constant 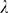 (in centimeters). The cable also expresses a voltage-dependent conductance with a gating variable 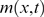 with activation function 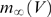 and time constant 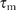 (in milliseconds). The equations governing the membrane potential 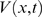 and the gating variable 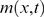 along the cable (excluding the oscillators) are 
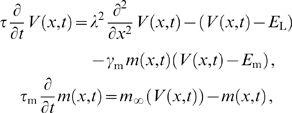
(9)where 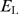 is the leak reversal potential, 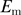 is the reversal potential of the active current, and 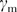 is the ratio of the maximal conductance of the active current to the leak conductance. The two oscillators form the periodically forced end conditions of the cable: 
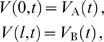
(10)with 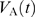 and 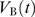 being the voltage traces of the two oscillators A and B that evolve according to 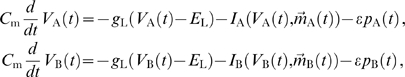
(11)where 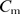 is the membrane capacitance (in 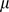
F/cm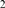), 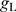 is the leak conductance (in mS/cm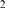), 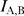 summarizes the voltage-dependent membrane currents generating the oscillations with the vector of gating variables 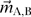 given by standard kinetic equations (e.g. see Equations 28 and 29). The terms 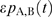 describe the perturbing currents from the cable to each oscillator with the small parameter 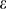 denoting the coupling. For a cable with diameter 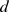 (in cm) and oscillators with membrane surface area 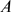 (in cm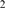), 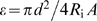, where 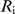 is the intracellular resistivity of the dendritic cable (in k
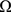
cm). The functions 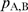 are given by 
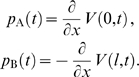
(12)


#### Determining the perturbations from the cable to the oscillators

In order to determine the perturbations 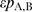 in Equation 11, we need to solve Equation 9 with the boundary conditions from Equation 10. To do so, we linearize Equation 9 about the membrane potential 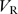 to which the cable would relax if it was not driven by the oscillators, yielding the quasi-active approximation for the cable [29],[30]. This approximation is appropriate as long as the voltage fluctuations around 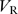 are sufficiently small. We define 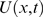 as the difference between the oscillating solution and the resting membrane potential 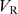, i.e. 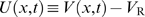 and we define 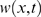 analogously as 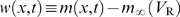. The equations describing the quasi-active cable now read 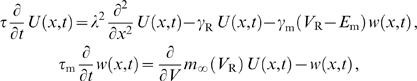
(13)where 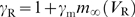 is the total membrane conductance of the cable at 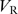 divided by the cable's membrane leak conductance.

The oscillators determine the voltage of the cable at 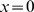 and 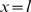. These voltages would need to be computed by solving the full system of equations for the dynamics of each oscillator, however since we consider weak coupling (meaning that the trajectories are only weakly perturbed by the cable currents) we can make use of the fact that the trajectories are periodic. Hence we expand 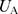 and 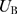 in a Fourier series, allowing for a possible phase difference 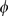 (in radians) between the oscillators: 
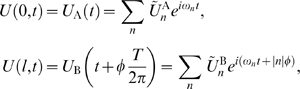
(14)where 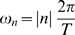, 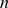 is an integer, 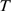 is the intrinsic oscillator period, and membrane voltages 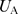 and 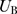 (in mV) are measured relative to 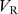.

The solution of the cable Equation 13 will also be periodic and we can write the equation in the frequency domain as 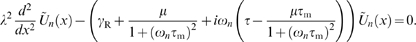
(15)


Using the boundary conditions defined by Equation 14 yields the solution: 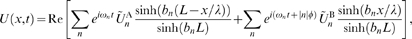
(16)where 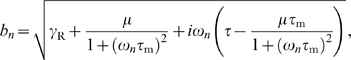
(17)with 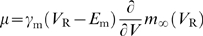 and 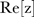 is the real part of the complex number 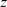. The parameter 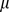 determines whether the active conductance that is present in the cable is regenerative (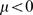), meaning that perturbations are amplified (e.g. a persistent sodium current), or restorative (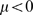), meaning that the active conductance counteracts perturbations from 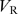 (e.g. the hyperpolarization activated inward current). As mentioned above, the perturbations that the oscillators receive from the cable is proportional to the derivative of the voltage with respect to 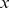. For the oscillator at 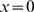 the perturbation from the cable is 
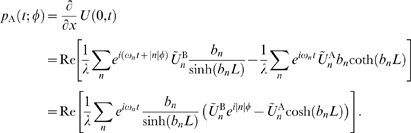
(18)


The perturbation from the cable at 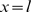 can be derived in the same way.

#### Phase description and interaction function

We have now derived the perturbations that an oscillator receives depending on the phase difference 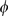 between the oscillators. In order to complete our analysis, we also need to compute how these perturbations act back on the phases of the two oscillators and thus on the phase difference. Each of the oscillators is described explicitly by a system of equations determining the dynamics of its voltage Equation 11. However, if we assume that the periodic solutions of such a system of equations are sufficiently attractive and the coupling is sufficiently weak we can write an equivalent phase model, see [24]. The phases of the two dendritic oscillators, 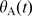 and 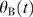 (in radians), evolve as
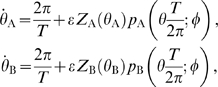
(19)where 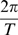 is the intrinsic oscillator frequency. The second term describes the effect of the cable on the phase. 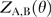 are the infinitesimal phase response functions of the respective oscillators and describe how much their phases are advanced or delayed in response to an infinitesimally small and short perturbation.

Since we consider weak interactions between the oscillators, 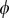 changes slowly with respect to the oscillation period. Therefore we can average the interaction between the oscillators (i.e. the products 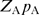 and 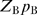 in Equation 19) over a cycle and obtain the interaction functions 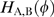. 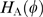 describes the average effect on the phase of oscillator A over one cycle as a function of 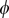: 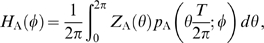
(20)with 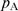 given by Equation 18. The interaction function 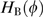 can be determined analogously. Note that with identical oscillators, we have 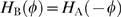.

### Interaction function for simplified dendritic oscillators

Consider identical oscillators when both 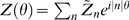 and 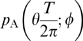 are dominated by the first Fourier component. One can show that the interaction function is given by 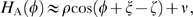
(21)where 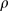 is a positive coefficient, 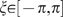 is a constant resulting from the cable filtering, 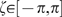 is a constant that results from the specific properties of the oscillators and 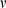 is a constant (see figure 1A). The expressions for the parameters are 
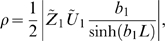
(22)

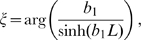
(23)

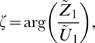
(24)

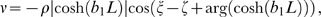
(25)where 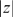 and 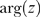 are, respectively, the absolute value and the angle of the complex number 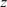.

#### Scaling of 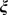 with 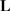


When 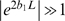 one can approximate 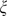 from Equation 23 by 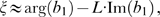
(26)where 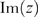 is the imaginary part of the complex number 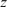, while making sure that 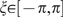.

#### Scaling of 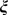 with membrane resistance 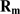


The membrane resistance 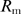 affects both the membrane resistance and the electrotonic length: 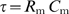 and 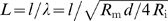, where 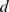 is the diameter of the cable, 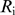 is the intracellular resistivity and 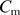 is the membrane capacitance. For small 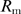 the imaginary part of Equation 17 vanishes and 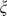 is zero from Equation 23. For large 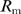, 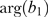 approaches 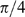 and the product 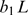 in Equation 23 tends to a constant proportional to 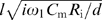; 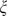 also saturates since it is equal to the sum of 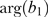 and 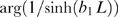.

#### Scaling of 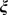 with oscillator frequency 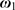


For low oscillator frequency 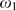, the value of 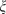 approaches zero as the imaginary part in Equation 17 goes to zero. With increasing frequency the term 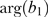 in Equation 26 approaches 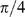, while the term 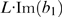 scales as the square root of the frequency. Hence for large 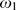, 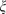 also scales as the square root of 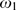.

#### Effects of active currents on 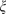


The effects of active currents on the phase-locking regimes can be seen from Equations 17 and 26: a regenerative current (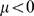) increases 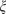 compared to a passive cable since it increases the imaginary part of the complex factor 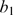. Equation 26 shows that therefore the ranges of 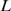 for the different phase-locking regimes shorten. In contrast, a restorative current (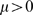) typically decreases the imaginary part of 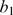 and therefore decreases 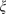, lengthening the phase-locking regimes. Note that for a range of frequencies 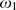, the imaginary part of 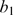 will change sign so that a restorative current can in fact make 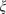 grow with increasing 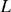 (see figure 2A).

For both restorative and regenerative currents the effects on 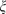 disappear for very high frequencies: the terms involving 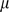 in Equation 17 go to zero. The only effect on 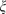 that remains is the decrease of the membrane resistance that results from the addition of the active current to the cable membrane (expressed in 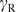).

### Skew of interaction function

The shape of the interaction function 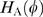 is determined by Equations 17, 18 and 20. When the electrotonic separation 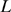 between the two oscillators goes to zero, we have a system of directly coupled oscillators and the interaction function 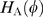 reduces to 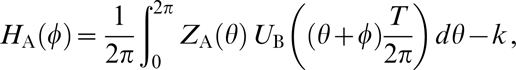
(27)where the constant 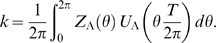.

Introducing an electrotonic separation 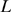 between the oscillators changes the shape of 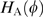 as a result of the cable filtering. When substituting Equation 18 into Equation 20 one sees that the symmetry of 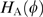 can only be affected by the 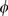-dependent term involving the voltage trace of oscillator B. As 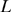 increases, the increasing cable filtering – determined by the absolute value of the term 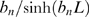 in Equation 18 – leads to dominance of a single Fourier component. Note that it is not necessarily the first Fourier component that will dominate. When 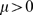 a higher order Fourier component can be the dominant one.

### Oscillator models

The equations for the Morris-Lecar type II oscillator [32] with parameters as in [40] read 
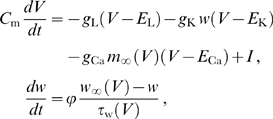
(28)with 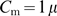 F/cm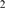, 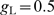 mS/cm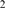, 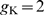 mS/cm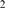, 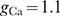 mS/cm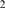, 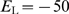 mV, 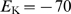 mV, 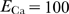 mV, 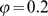, 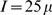
A/cm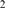, and where 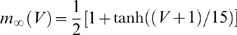, 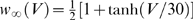, and 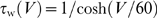.

The equations describing the subthreshold oscillator are of the same form as those used by Morris and Lecar [32]. The oscillatory dynamics emerge from the interaction between the persistent sodium current 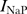 and the hyperpolarization activated inward current 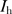. The current descriptions are based on the data from [33],[41]. The dynamics of 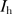 are described by a single gating variable 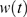 with activation function 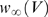 and time constant 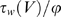 (in milliseconds). The voltage-dependent activation of 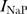 is described by 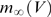 and is instantaneous. The equations read 
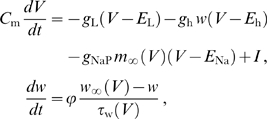
(29)with 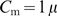
F/cm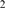, 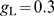 mS/cm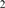, 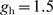 mS/cm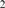, 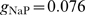 mS/cm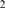, 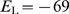 mV, 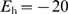 mV, 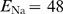 mV, 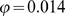, 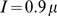
A/cm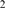, and where 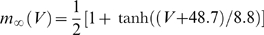, 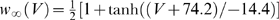, and 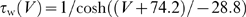.

### Numerical simulations

The numerical simulations for figure 4, 6 and 7 used Morris-Lecar type II oscillators and simulations for figure 5 used the subthreshold oscillator model described above. The cable was discretized into isopotential compartments with electrotonic length 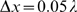. The perturbing currents from the cable to, for example, oscillator A are of the form 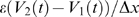 with 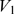 and 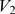 denoting the membrane potential of the first two compartments. The parameter 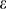 determines the coupling between the cable and the oscillators and is specified in the different figure captions. Simulations for figure 7 include a soma with an integrate and fire mechanism with a fixed threshold at 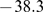 mV. When the threshold is reached a spike is generated with a 1 ms peak at 30 mV after which the somatic 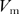 is reset to 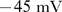 mV for 4 ms. The phase response curves were calculated by determining the system's adjoint [24].

## Supporting Information

Text S1 Direct compartmental simulations support the weak coupling assumption
(0.03 MB PDF)

Figure S1 Results from numerical simulations with a continuous cable model agree with weak coupling predictions. Voltage dependent conductances of the Morris-Lecar type II oscillators are inserted in the ends of a cable with diameter 1 micro;m, membrane capacitance C_m_ = 1 micro;F/cm^2^, intracellular resistivity R_i_ = 0.2 kΩ cm and membrane resistance R_m_ = 20 kΩ cm^2^. Panels A, B and C show the voltage trajectories recorded at the ends of the cable for an electrotonic distance between the active segments of 1.1, 2.1 and 1.5, respectively. Black bars denote perturbations of 100 ms duration to test for stability of the phase-locked state.
(0.89 MB EPS)
