# Supplementary material for: The Role of Ongoing Dendritic Oscillations in Single-Neuron Dynamics
Source: PLoS Comput Biol. 2009 Sep 4;5(9):e1000493. doi: 10.1371/journal.pcbi.1000493 (PMC2725317; doi:10.1371/journal.pcbi.1000493)
Supplement: Text S1 — Direct compartmental simulations support the weak coupling assumption (0.03 MB PDF) [file pcbi.1000493.s001.pdf]

Direct compartmental simulations support the weak coupling assumption

We simulated a cable in which we inserted the voltage-dependent conductances that underlie the Morris-Lecar type II oscillator in the end segments. This continuous cable model does not use the explicit assumption of weak coupling. The cable has diameter 1 μm, membrane capacitance $C_{\mathrm{m}} = 1\,\mu\mathrm{F/cm}^2$, intracellular resistivity $R_{\mathrm{i}} = 0.2$ kΩ cm, and membrane resistance $R_{\mathrm{m}} = 20$ kΩ cm^2 with reversal potential $E_{\mathrm{L}} = -50$. In order for the active segments to generate oscillations while using the same conductance densities as in the Morris-Lecar model (see Methods), the active segments needed to be at least 150 μm in length. Current I was injected into the active segments with the same current densities as in the Morris-Lecar model to produce oscillations. The leak conductance g_{L} in the active segments was decreased slightly to $g_{\mathrm{L}} = 0.3$ mS/cm^2. The cable was discretized into isopotential compartments with electrotonic length $\Delta x = 0.04\,\lambda = 20$ μm. The length of the passive stretch of cable in between the active segments was varied and the phase-locked solution was determined numerically. Stability of a phase-locked solution was tested by adding $0.1 - 0.6\,\mu\mathrm{A/cm}^2$ to one of the active segments for a duration of 100 ms (black bars above voltage traces in figure S1). Results from these simulations agree with our analytical predictions, showing synchronized phase-locking for small L, a bistable regime around $L \sim 1.5$ and anti-phase locking for larger L (figure S1).
